# Prevalence and correlates of depressive symptoms among people living with HIV attending tertiary care hospitals in Oman

**DOI:** 10.11604/pamj.2020.37.90.23294

**Published:** 2020-09-25

**Authors:** Abdullah Al Madhani, Lara Al Harthi, Abdullah Balkhair, Moon Fai Chan, Badar SR Albusaidi, Nasser Al Sibani, Samir Al-Adawi

**Affiliations:** 1 Psychiatry Residency Program, Oman Medical Specialty Board, Muscat, Oman,; 2 Department of Behavioural Medicine, College of Medicine and Health Sciences, Sultan Qaboos University, Muscat, Oman,; 3 Infectious Diseases Unit, Department of Medicine, Sultan Qaboos University Hospital, Muscat, Oman,; 4 Department of Family Medicine and Public Health, Sultan Qaboos University, Muscat, Oman,; 5 Department of Nursing/Infectious Diseases Unit, Sultan Qaboos University Hospital, Muscat, Oman

**Keywords:** Depression, HIV, people living with HIV, psychology, Oman

## Abstract

**Introduction:**

depressive symptoms have been widely reported among people living with HIV (PLHIV) around the world. The extent of this on PLHIV in the Arabian Peninsula is unclear. This study aims to examine the prevalence of depressive symptoms using the Patient Health Questionnaire-9 (PHQ-9) in adult Omani patients with HIV attending a tertiary care hospital. It also aims to investigate the relationship between clinical and socio-demographic variables and depressive symptoms.

**Methods:**

LHIV, age >18 (n=101), participated in the study who were followed up at a teaching hospital in Muscat, Oman. Participants were identified by a convenient and consecutive sampling of eligible patients who came for consultation on the days that the investigator conducted recruitment. The presence of depressive symptoms was quantified by the Patient Healthcare Questionnaire-9 (PHQ-9). Various socio-demographic backgrounds and risk factors will be also sought as well as activities of daily living (ADL). Chi-square test, Fisher´s exact test, t-test and logistic regression were used to explore which variables were associated with patients having depressive symptoms.

**Results:**

the prevalence rate of depressive symptoms in this cohort was 41.6%. Depression among PLHIV was found to be significantly associated with age (p <0.001), HIV disease duration (p <0.001), total dependency for ADL (p <0.001) and comorbid hypertension (p <0.001).

**Conclusion:**

depressive symptoms are common in Omani patients living with HIV. HIV care providers are urged to be vigilant to recognize depressive symptoms in these mood-vulnerable populations and to call for a multidisciplinary team with mental health professionals, for the prevention and treatment of depressive symptoms among PLHIV in Oman.

## Introduction

The pandemic of the Human Immunodeficiency Virus (HIV) and Acquired Immunodeficiency Syndrome (AIDS) is widely established to constitute a global challenge [1], and an Eastern Mediterranean Region country such as Oman, is of no exception. However, Oman is categorized by the World Health Organization (WHO) as a low prevalence country for HIV with a rate of <1% and a total of 3,025 adult Omani patients diagnosed with HIV as of 2018, of which 1,532 individuals were alive [2]. There are a plethora of studies highlighting various clinical manifestations of HIV/AIDS [3,4], while some studies have also reported awareness of HIV/AIDS [5]. Overall, Oman is known to be as having a low-prevalence epidemic [6] and the country has embarked on significantly reducing fatalities associated with HIV/AIDS [4]. While Oman has been able to mitigate HIV/AIDS attributed mortality largely through the availability of effective antiretroviral therapy [7], several other aspects of the care for PLHIV such as comprehensive psychosocial treatment require more efforts. Various studies have suggested that living with HIV/AIDS poses a major psychological burden on PLHIV, largely due to the chronicity and complications of the disease, the fact that HIV is perceived to be a life-living condition with no cure, as well as the stigma it involves [8].

Another debilitating and intransigent sequel affecting PLHIV is an afflictive emotion known as depression. Epidemiological studies carried in different populations around the world suggest that debilitating and afflictive emotion or clinical depression affects approximately 3% of the population around the world [6]. The magnitude and impact of depressive disorders have been predicted that it will surpass the current ‘enemy of health´ such as cancer and cardiovascular diseases in terms of mortality, dependency and disability. In the year 2020, the depressive symptoms will be the second leading cause of disease [9]. Depressive symptoms have traditionally been thought to be an episodic disorder; however, recent evidence suggests that depressive illnesses have a chronic course and are often associated with dependency and disability. Just like depression, HIV/AIDS has been projected to be the world´s number two leading cause of disability by 2030 [10]. There is wide variation in the presence of depressive symptoms in PLHIV. Some studies have estimated the prevalence to vary from 22% to 71% [11-13]. It has been widely acknowledged that depression in PLHIV is not simply feeling ‘blue´, but rather a strong predictor for heightening health care utilization [14,15], denting the quality of life [16] and increases the threat for ‘risk behavior´ that may heighten the likelihood of HIV transmission [17,18].

Other factors associated with depressive symptoms in PLHIV include poor adherence to antiretroviral therapy leading to virologic, immunologic and clinical failure [19]. The presence of depressive symptoms has been suggested to trigger negative biopsychosocial functioning which, in turn, has ramifications in HIV/AIDS-related outcomes. To the best of our knowledge, no published studies are examining depressive symptoms in PLHIV in Oman or the Arabian Gulf region. This study aims to explore the prevalence of depressive symptoms among PLHIV in Oman, to explore the relationship between clinical and socio-demographic and depressive symptoms. If depressive symptoms are common in PLHIV in Oman, then the natural extension of this is to include interventions to mitigate the severity of affective disorders in this population. The findings from this study have the potential to lay the groundwork for identifying predictors of emotional problems in PLHIV, so that appropriate health education measures could be considered and planned. The findings from this study may be extended to the care of other chronic diseases with much needed ‘paradigm shift´ in the philosophy of healthcare in Oman, where the focus has been geared towards ‘preserving life´ without consideration to the quality of life that entailed.

## Methods

**Setting**: the study was conducted in the infectious disease clinic at Sultan Qaboos University Hospital, 480-bed teaching and referral hospital located in the nations´ capital, Muscat. The clinic is one of the referral centers for PLHIV in Oman for over 25 years.

**Sample calculation:** the required sample size was calculated using EPi Info Software after setting the power at 80%, precision between 7% and 8% and confidence interval (CI) at 95%. Considering the prevalence rate of depression among PLHIV in published literature is 20% [20] and the number of patients with HIV seen over three months is approximately 140, the required sample size was determined to be from 84 (precision=8%) to 105 (precision=7%) to calculate the period prevalence.

**Recruitment of participants:** the systemic random sampling approach was adopted to recruit participants. The sampling frame (the patients who were seen over three months) list was accessed with the aid of the ‘track system´/medical record (Hospital Information System). The sampling fraction (sampling frame/required sample size) was identified to be approximately 3. Then the first participant to be recruited was identified randomly from the first 3 patients in the list (sampling fraction) using software randomizer. It was found to be the 2^nd^ patient on the list. The recruitment sequence has been as follows: 2^nd^, then 5^th^, then 8^th^ etc. This protocol prevailed until the study reaches the required sample size. This study abides by the World Medical Association´s Declaration of Helsinki (1964-2008) for Ethical Human Research including confidentiality, privacy and data management [21].

**Inclusion/exclusion criteria**: adult patients (age >18 years) with a diagnosis of HIV of more than six months attending the clinic were included in the study. Patients with cognitive, emotional, or behavioral disorders that would prevent them from answering the study questionnaire were excluded from the study.

### Outcome measures

**Depressive symptoms:** the Patient Health Questionnaire-9 (PHQ-9) was used to detect the presence of depressive symptoms. The progenitor of PHQ-9 has specifically phrased items to capture the criteria for major depressive disorder as the Diagnostic and Statistical Manual of Mental Disorders, 4^th^ edition (DSM-IV) [22]. The PHQ-9 is utilized to identify the presence of depression in various clinical populations including PLHIV [23]. PHQ-9 was previously validated in the Omani population and accordingly a cut-off score of 10 was deemed to be most suitable to differentiate case and non-case [24].

Social-demographic background and risk factors: in addition to the investigation of the presence and absence of depressive symptoms, background variables including age, gender, marital status and educational level will be sought from the participants. Housing status and monthly income were used as surrogates for the socio-economic status. A personal and family history of depression was also investigated. Clinical variables including disease duration, adherence to antiretroviral therapy, virologic and immunologic responses as reflected by viral load and CD4 cell count were collected.

Activities of daily living: functional and self-directed activities were examined using the Arabic-version of activities of daily living [25], Katz Index of Independence in Activities of Daily Living (KIADL) [26]. The participants are required to state ‘yes´ or ‘no´ in self-sufficient on six domains such as bathing, dressing, toileting, transferring, continence and feeding. KIADL is scored as follows: ‘totally independent´, ‘mild dependent and ‘total dependent´. For the present purpose, participants were categorized as either total ‘minimally to total independent´ vs ‘total dependent´.

Data analysis: data were analyzed using Statistical Package for Social Sciences, SPSS 23.0 (IBM SPSS® Inc. Chicago, IL, USA). The results of those associated with depressive symptoms were displayed using descriptive statistics. The participants that scored ≥10 on the PHQ-9 were considered as depressed. First, univariate analysis was used and social-demographic and clinical variables were evaluated with the Chi-square test, Fisher´s exact test, or t-test to reveal association on the depressed and non-depressed group. Following this, logistic regression analysis was used where depression was the dependent variable and variables considered significant in the univariate analysis were the independent variables. The level of significance was set at 5%. This analysis could then address the research aim of identifying the contributing variables associated with depression.

**Ethical approval and consent to participate:** all procedures performed in studies involving human participants were following the ethical standards of the institutional (Medical Research Ethics Committee, College of Medicine and Health Sciences, Sultan Qaboos University (SQU-EC/028/18, MRC #1660)) and with the 1964 Helsinki declaration and its later amendments or comparable ethical standards. All participants provided written informed consent.

## Results

The present study was conducted on 101 adult patients with a diagnosis of HIV. Using the PHQ-9, the prevalence rate of depression in this cohort was 41.6% (n=42). As shown in Table 1 below, 64.4% (n=65) of patients were male, with an average age of 40.7 years (SD=9.1). Furthermore, 80.2% (n=81) of PLHIV had secondary level education or below, with 76.2% (n=77) that were residing in their own houses. Also, 62.4% (n=63) and 79.2% (n=80) reported the absence of depressive symptoms in self and family respectively. Significant associations were found only between depressive symptoms and age (t=9.810, p <0.001), personal history of depression (p=0.010), and family history of depression (p=0.009). As shown in Table 2, in clinical outcomes, a significant association was found between depressive symptoms and disease duration (p<0.001), total dependency for activities of daily living (p<0.001), hypertension (p <0.001), and diabetes mellitus (p=0.018). In Table 1 and Table 2, in the univariate analysis, age, personal and family history of depression, HIV duration, total dependency for activities of daily living, hypertension and diabetes mellitus, were significantly associated with depressive symptoms. As such, these seven risk factors were included in the logistic regression model for further analysis. The analysis showed that older patients (p=0.001) and those with a longer duration of HIV (p=0.029), were more likely to have depressive symptoms. PLHIV who have hypertension were 19 times (OR=19.528, p=0.012) more likely to endorse depressive symptoms than normotensive patients. PLHIV who reported total dependency for activities of daily living were 204 times more likely (OR=204.158, p=0.001) to have depression than PLHIV who reported minimal dependency or were independent.

**Table 1:** univariate and multivariate (logistic regression) analysis for depressive symptoms and sociodemographic background in people living with HIV seeking consultation at tertiary care in Oman

			Depression (PHQ-9 >10)					
		Total=101	Yes (n=42)	No (n=59)	Univariate		Multivariate^	
Measurement		n (%)	n (%)	n (%)	OR	p-value	OR	p-value
Socio-demographic								
Gender	Male	65 (64.4)	29 (69.0)	36 (61.0)	1.425	0.406		
	Female	36 (35.6)	13 (31.0)	23 (39.0)				
Age (years)	Mean±SD	40.7±9.1	48.3±7.2	35.4±5.9	9.810^c^	<.001	0.876	0.001*
	Median [Range]	40.0 [19.0-40.0]	49.0 [19.0-60.0]	37.0 [19.0-45.0]				
Marital status	Single	31 (31.7)	10 (23.8)	22 (37.3)	0.526	0.151		
	Not single	69 (68.3)	32 (76.2)	37 (62.7)				
Educational level	Secondary or below	81 (80.2)	31 (73.8)	50 (84.7)	0.507	0.209		
	College or above	20 (19.8)	11 (26.2)	9 (15.3)				
House	Rent	24 (23.8)	6 (14.3)	18 (30.5)	0.380	0.096		
	Owned	77 (76.2)	36 (85.7)	41 (69.5)				
Monthly income (OMR)	<400	51 (51.0)	17 (40.5)	34 (58.6)	5.774	0.131		
	400-799	23 (23.0)	12 (28.6)	11 (19.0)				
	800-1199	14 (14.0)	5 (11.9)	9 (15.5)				
	1200+	12 (12.0)	8 (19.0)	4 (6.9)				
Personal history of depression	Yes	38 (37.6)	22 (62.4)	16 (27.1)	2.966	0.010*	0.143	0.191
	No	63 (62.4)	20 (47.6)	43 (72.9)				
Family history of depression	Yes	21 (20.8)	14 (33.3)	7 (11.9)	3.714	0.009*	12.803	0.119
	No	80 (79.2)	28 (66.7)	52 (88.1)				

a t-test (t-statistics), OR, Odds Ratio; *, sig. at p<0.05; PHQ-9, the Patient Health Questionnaire-9, ^ Logistic adjusted by age, subject is on ART, personal and family history of depression, duration of HIV, activity of daily living, Diabetes Mellitus and hypertension in univariate analysis, Hosmer and Lemeshow Test, χ^2^=14.305, p=0.074; Sensitivity=90.5%, Specificity=94.9%, overall=93.1%

**Table 2:** univariate and multivariate (logistic regression) analysis for depressive symptoms and clinical features in people living with HIV seeking consultation at tertiary care in Oman

Measurement			Depression (PHQ-9 >10)					
		Total=101	Yes (n=42)	No (n=59)	Univariate		Multivariate^	
		n (%)	n (%)	n (%)	OR	p-value	OR	p-value
Clinical outcome								
On ART^a^	Yes	84 (83.2)	37 (88.1)	47 (79.7)	1.889	0.264		
	No (newly diagnosed)	17 (16.8)	5 (11.9)	12 (20.3)				
Duration of illness (year)	Mean±SD	8.1±5.2	10.6±3.9	6.3±5.2	4.525^c^	<.001*	0.825	0.029*
	Median [Range]	7.0 [1.0-29.0]	10.0 [4.0-22.0]	4.0 [1.0-29.0]				
Activity of daily living	Total dependency	31 (30.7)	29 (69.0)	2 (3.4)	63.577^b^	<.001*	204.158	0.001*
	Minimum to totally independent	70 (69.3)	13 (31.0)	57 (96.6)				
Co-morbid-osteoporosis	Yes	5 (5.0)	3 (7.1)	2 (3.4)	2.192^b^	0.647		
	No	96 (95.0)	39 (92.9)	57 (96.6)				
Co-morbid-hypertension	Yes	16 (15.8)	14 (33.3)	2 (3.4)	14.250^b^	<.001*	19.528	0.012*
	No	85 (84.2)	28 (66.7)	57 (96.6)				
Co-morbid-hyperlipidemia	Yes	16 (15.8)	9 (21.4)	7 (11.9)	2.026	0.194		
	No	85 (84.2)	33 (78.6)	52 (88.1)				
Co-morbid-diabetes mellitus	Yes	20 (19.8)	13 (31.0)	7 (11.9)	3.330	0.018*	0.778	0.820
	No	81 (80.2)	29 (69.0)	52 (88.1)				

a ART - Antiretroviral Therapy; ^b^Fisher's Exact Test (χ^2^ statistics); ^c^t-test (t-statistics), OR, Odds Ratio; *, sig. at p<0.05; PHQ-9, the Patient Health Questionnaire-9, ^ Logistic adjusted by age, subject is on ART, personal and family history of depression, duration of HIV, activity of daily living, Diabetes Mellitus and hypertension in univariate analysis, Hosmer and Lemeshow Test, χ^2^=14.305, p=0.074; Sensitivity=90.5%, Specificity=94.9%, overall=93.1%

## Discussion

To our knowledge, this is the first study on the prevalence of depressive symptoms among PLHIV in Oman. This study finds that the psychological burden of living with HIV in Omani society is immense, with 42% of patients suffering from depressive symptoms according to the Patient Health Questionnaire-9 (PHQ-9). Our findings are consistent with several other studies [27]. In a recent systematic review and meta-analysis of depression in PLHIV in low, middle and high-income countries, it is suggested that 12.8% to 78% of patients have depressive symptoms. One of the plausible explanations for this wide variation in reporting of depressive symptoms is the tool used to assess depression in these studies; PHQ-9 versus the gold-standard patient interview. It is warranted, therefore, that future studies could examine the presence of depression among PLHIV in Oman using the gold-standard interview. Another probable explanation is the variation in the sociodemographic status of the societies where the studies were performed. This study finds that patient age plays a significant role in influencing endorsement of depressive symptoms. Contrary to societal belief, this study suggested that younger age (35.4±5.9 cohort), is protective against succumbing to depressive symptoms. It is known that depressive symptoms in the general population are more prevalent among the elderly [28]. Although there is a dearth of studies that explore the relationship between age and depression in PLHIV [13,29,30], the exact reason(s) for the tendency of older PLHIV in this study to suffer from depression is unclear.

Another interesting finding from this study is the relationship between the duration of living with HIV and the experience of depressive symptoms. The study finds that patients with a long history of HIV tend to suffer from depression in comparison to those with relatively recent diagnoses. Contrariwise, in studies on grief and mourning, bereaving individuals tend to undergo depressive symptoms upon the diagnosis of life-limiting diseases such as HIV/AIDS but eventually adjust to the ‘bad news´ [31]. Whether this unexpected finding is influenced by concurrent ‘aging´, societal factors, or other confounders, is unclear. Furthermore, this study did not explore whether depression in this cohort is temporary or long-lasting and therefore, chronic. Further studies should be conducted to examine whether depressive symptoms are heightened immediately upon diagnosis or subsequently fade-out. Until then, the finding from this study suggests that older patients with HIV and patients with a long duration of living with HIV shall be targeted for mental health interventions. Depressive symptoms in PLHIV have been shown in several studies to negatively impact the link to and retention in care and adherence to antiretroviral therapies [32,33]. In addition, depression is also associated with poor adherence to lifestyles that have the potential to mitigate distress and enhance wellbeing [8].

In this study, the majority of participants (n=84, 83%) were on antiretroviral therapy (ART) and when surveyed, (n=37, 44%) endorsed depressive symptoms. However, five out of the newly diagnosed 17 patients who were not on ART when surveyed (29%) reported depressive symptoms. We postulate that exposure to long-term ‘non-curative´ ART, enforces patients´ sense of hopelessness leading to depressive symptoms. Moreover, most surveyed patients in antiretroviral therapy were on Efavirenz, an ART agent that is notorious for causing neuropsychiatric unwanted effects. However, none of these hypotheses were tested in the present study. Impairment in functionality including the ability to perform activities of daily living (ADL), is a well-recognized sequela of life-limiting diseases including advanced HIV and its´ associated complications. This was examined using the Katz Index of Independence in Activities of Daily Living (KIADL) [26]. This study finds that 30.7% of the cohort (n=31) had a problem with self-directed and proactive behavior. Where 93% of this total dependency group endorsed depressive symptoms in comparison with 19% who reported minor to total independence, indicating a significant relationship between total dependency in ADLs and depressive symptoms (OR=204.158, p=0.001). This strong association is consistent with findings from a previous study - albeit not conducted in PLHIV [34].

It is unclear whether depressive symptoms in the cohort examined in the present study are a cause or effect. It is known that depressive symptoms tend to strongly feature a lack of hedonic tone as well as impaired motivation. It is also possible that failure to have adequate independence in ADL may render one as having an intransigent illness which in turn, leads to afflictive emotion. The present study did not examine this relationship. Metabolic syndromes such as osteoporosis, hypertension, hyperlipidemia and diabetes mellitus, are frequent ART-associated comorbidities in PLHIV. The present study attempted to explore the association (if any) of these comorbidities with depression. Except for hypertension, none of the other three conditions were found to have an association with depressive symptoms. We have no plausible explanation for this interesting observation. However, we hypothesize that there are two views regarding this association. One view is that hypertension is likely to be secondary to depressive symptoms as suggested by a previous study [35]. The second suggests that chronic use of ART tends to be associated with hypertension [36] and in the present study, the majority of participants (83.2%) were recorded to have been on ART - therefore the role of ART seems plausible. This study, as well as others, suggest that the presence of depressive symptom is strongly associated with developing hypertension. Mitigating depressive symptoms and hypertension should be part of the algorithm of care for PLHIV since there is an increase in the risk for stroke and ischemic heart disease [35].

**Strengths and limitations of this study:** this study had several limitations. First, PLHIV in this study was receiving multifaceted care in a specialized teaching hospital setting, hence generalizations of the study findings to other settings may not be possible. Second, the tool used to identify depressive symptoms in this study - Patient Health Questionnaire-9; PHQ-9 - is not considered the gold-standard method. However, this method has been widely used to identify the presence of depressive symptoms in other patient populations. Future studies should employ an instrument for taxing mood disorders that are sensitive to PLHIV. Third, the present study did not examine the interplay between depressive symptoms and clinical, virologic, or immunologic responses in PLHIV. Disease outcome is expected to influence patient mood and behavior. Future studies overcoming these limitations are much needed.

## Conclusion

This study has embarked to explore the prevalence of depressive symptoms among PLHIV. Depressive symptoms are prevalent in Omani patients living with HIV, where the present data indicated 42% as endorsing depressive symptoms. The related aim is to determine the factors that are associated with depression. Duration of living with HIV, increasing age, dependency for activities of daily living and the presence of co-morbid conditions such as hypertension, are key to experiencing depressive symptoms in this stigmatized and fragile population. If future studies would confirm the present findings, it would be essential for healthcare infrastructure to contemplate the best practice in identifying and mitigating depressive symptoms in this population. It is also recommended for HIV care providers to practice vigilance in identifying depressive symptoms in their patients and seek specialized referral when appropriate. Addressing such unmet needs would further heighten the quality of life of PLHIV. We concur with ‘no health without mental health´.

### What is known about this topic

- Depressive symptoms occur in 12.8% to 78% of people living with HIV (PLHIV);
- Depressive symptoms in PLHIV tend to trigger adverse HIV/AIDS-related outcomes;
- Recognition of depressive symptoms in PLHIV from Eastern Mediterranean Region

### What this study adds

- Depressive symptoms occur at 41.6% among PLHIV in Oman;
- Covariate of depressive symptom include age, disease duration and comorbid hypertension;
- Depressive symptoms reduce one efficiency in activities of daily living.
